# Middle East respiratory syndrome coronavirus (MERS-CoV) internalization does not rely on DPP4 cytoplasmic tail signaling

**DOI:** 10.1038/s44298-024-00080-y

**Published:** 2024-12-30

**Authors:** Karthika Thankamani, Divakar Shubham, Gayatri Kandpal, Ann Mary Isaac, Modenkattil Sethumadhavan Kavitha, V. Stalin Raj

**Affiliations:** Virology Scientific Research (VSR) Laboratory, School of Biology, Indian Institute of Science Education and Research Thiruvananthapuram (IISER-TVM), Thiruvananthapuram, Kerala India

**Keywords:** Virus-host interactions, SARS virus

## Abstract

Middle East respiratory syndrome coronavirus (MERS-CoV) infects respiratory epithelial cells in humans and camels by binding to dipeptidyl peptidase 4 (DPP4) as its entry receptor. DPP4 is a multifunctional type II membrane protein with a long ectodomain and a short six-amino-acid (aa) cytoplasmic tail. MERS-CoV is known to bind to the ectodomain of DPP4 to gain entry into the host cell. However, the role of the cytoplasmic tail in the entry process remains unclear. Here, we show that mutating or deleting individual aa residues or the entire cytoplasmic tail of DPP4 (ΔcytDPP4) does not completely prevent DPP4 from being inserted into the membrane or from allowing the binding of the MERS-CoV spike protein and pseudovirus infection. Although two mutants, ΔcytDPP4, and a single aa deleted DPP4 (ΔK6DPP4) displayed less surface presentation than wtDPP4, the spike protein could still bind and localize on different DPP4 mutants. The reduced surface expression of ΔK6DPP4 might be due to the extended transmembrane domain, which is altered by the hydrophobic tryptophan (W) residue adjacent to the deleted K6. Furthermore, HEK293T cells transiently expressing DPP4 mutants were permeable to MERS-CoV pseudovirus infection. Not only transiently expressing cells but also cells stably expressing the ΔcytDPP4 mutant were susceptible to MERS-CoV pseudoviral infection, indicating that the DPP4 cytoplasmic tail is not required for MERS-CoV entry. Overall, these data suggest that, although MERS-CoV binds to DPP4, other host factors may need to interact with DPP4 or the spike protein to trigger internalization.

## Introduction

MERS-CoV belongs to the family Coronaviridae and has a positive-sense, single-stranded RNA genome of 30 kb in size^1^. It causes mild to severe lower respiratory tract infections in humans. The clinical signs include cold-like symptoms, fever, cough, and pneumonia, often leading to death^2^. The first MERS-CoV outbreak was reported in Saudi Arabia in 2012^3^, and subsequent cases were reported in approximately 27 countries, but the majority of the cases have been reported in the Middle East region^4^. As of May 2024, the World Health Organization reported more than 2500 infected cases globally, including 943 deaths^5^. Dromedary camels are proposed to be the intermediate host for this virus, as MERS-CoV neutralizing antibodies have been detected in camels^6^. Closely related viruses isolated from camels from the Middle East, Southern Africa, and Egypt are identical to human isolates, and these viruses are permeable to human cells^7–9^. In addition, camel workers became positive for MERS-CoV after exposure to camels, and the virus isolated from both patients and camels was similar^7^. Moreover, young camels shed a high viral load on their nasal cavity due to the presence of high levels of MERS-CoV receptors in the respiratory tract of this species^10,11^. The presence and distribution of the MERS-CoV receptor in the respiratory tract correlate with virus transmission, implying that frequent spillovers from this species to humans may occur in the near future^12^.

MERS-CoV uses dipeptidyl peptidase 4 (DPP4) as an entry receptor^13^, which belongs to the serine peptidase family and is predominantly expressed in epithelial cells of the lungs, liver, kidney, intestine, thymocytes, and alveolar macrophages^14^. Its physiological function is associated with cleaving peptides^15^ that regulate glucose metabolism^16–18^ and T-cell activation^19,20^. The distribution of DPP4 in host cells plays a vital role in the transmission of MERS-CoV between camels and humans, as well as the severity of the infection^21^. In particular, the human lower respiratory tract shows a higher expression of DPP4, which can result in an increased severity of infection but also serves as a limiting factor for transmission^22^. In contrast, the nasal cavity and small intestine of camels showed a high expression of DPP4, which has the potential to enhance the cross-species transmission of MERS-CoV^10^. Similarly, other human coronaviruses use distinct membrane receptors for their entry into the host cells. For example, HCoV-229E uses aminopeptidase N (APN)^23^ and SARS-CoV-1, SARS-CoV-2, and HCoV-NL63 use angiotensin-converting enzyme 2 (ACE2)^24–26^. In contrast to DPP4, ACE2 is predominantly expressed in the upper respiratory tract, which helps the virus attach and replicate in the nasal epithelial cells^27^. The presence of ACE2 in the upper respiratory tract of humans explains how SARS-CoV spreads between humans and why SARS coronavirus transmission is faster than other coronaviruses^28^.

The infection cycle of coronaviruses begins with the binding of virus spike glycoprotein to the receptor on the host cell surface, which triggers receptor signaling and allows the virus to internalize and enter the cells^29,30^. Post-binding of the virus to its receptor, the cytoplasmic domain of the receptor often plays a key role in triggering the internalization of the virus-receptor complex into the host cell^31–33^. These internalization processes might be triggered by the primary receptor, a co-receptor, or other host factor(s) that recruit host proteins to facilitate internalization. HIV, for example, uses CD4 as its primary receptor, but successful internalization and infection require the presence of other co-receptors, such as CCR5 and CXCR4^34,35^. Binding to CCR5, together with CD4, causes conformational changes that allow the viral membrane to fuse with the host cell membrane, resulting in internalization^36^. The influenza virus attaches to sialic acid-containing receptors on the cell surface^37^. Host factors, including clathrin, which is recruited to the receptor binding site, aid in the internalization process^38^. In the case of SARS-CoV-1 and -2, post-binding to ACE2, the cytoplasmic domain does not trigger the internalization process^39^ and the entry might be triggered by other cellular machineries. However, despite molecular and structural insights into MERS-CoV and the role and involvement of other host factors in entry, understanding of how DPP4 signaling pathways actively drive virus/receptor complex internalization is limited. MERS-CoV internalization might be triggered by DPP4 alone or along with other host factors such as sialic acid^40^, tetraspanin 9^41^, host proteases TMPRSS2/4^42,43^, or other unknown host factor(s) to contribute to virus internalization. In contrast to ACE2, DPP4 has only six amino acids in its cytoplasmic tail and no endocytosis motif other than a putative phosphorylation site. Nevertheless, DPP4 functions in the IKK-NFκB signaling pathway and has been reported to interact with an intracellular protein called CARMA1 through its cytoplasmic tail, leading to T cell activation^44^. However, no other functions of DPP4 cytoplasmic tail have been discovered yet. Therefore, we hypothesized that the DPP4 six amino acid long cytoplasmic tail signaling plays a role in MERS-CoV entry. Using bioinformatic analysis and mutagenesis followed by functional studies, we showed the role of the cytoplasmic tail of DPP4 in MERS-CoV entry.

## Results

### MERS-CoV entry is mediated by two distinct entry pathways

**Fig. 1:**
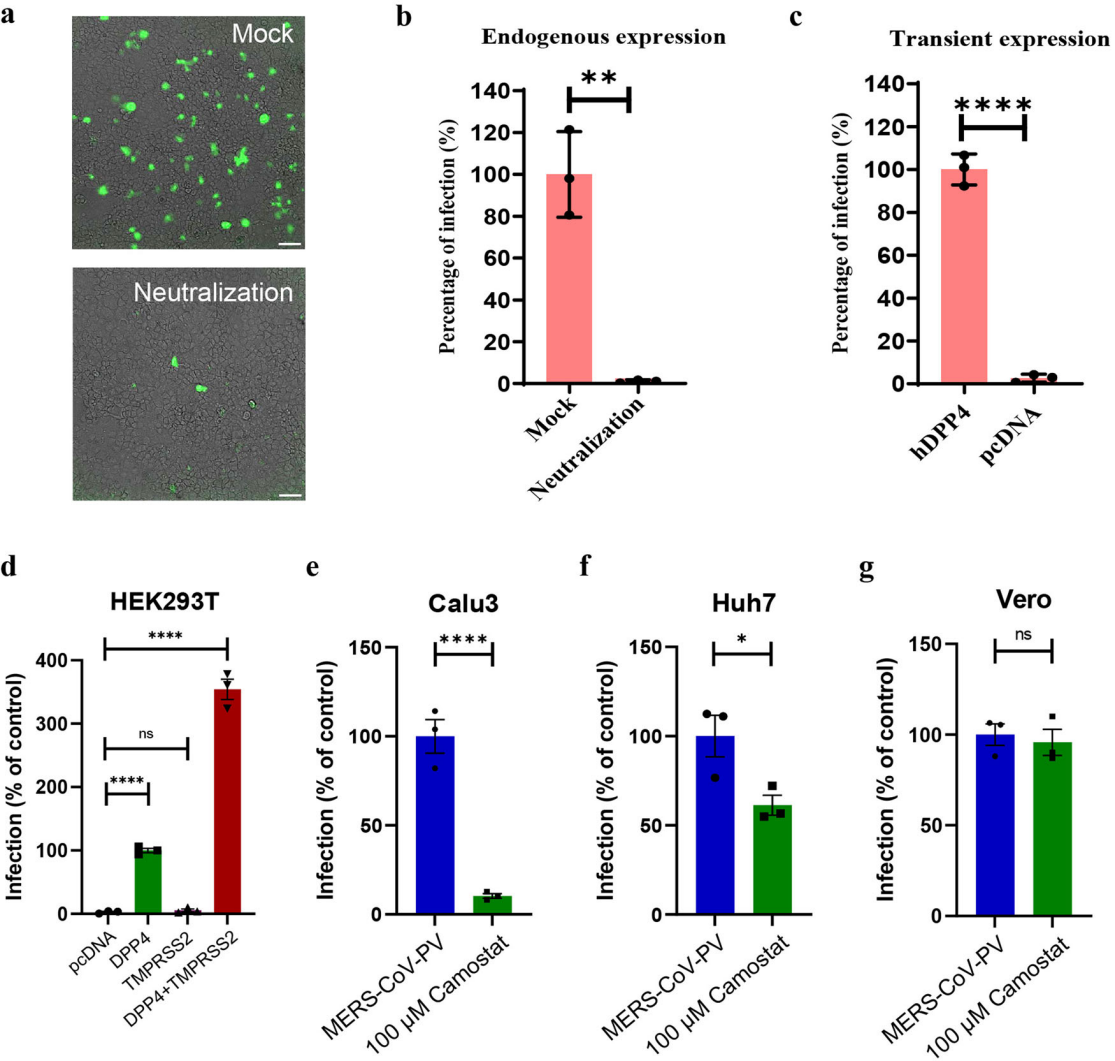
MERS-CoV entry is mediated by two distinct entry pathways. **a** Representative brightfield fluorescence image and a corresponding graph showing a reduction in the percentage of infection of MERS-PV-GFP in Huh7 cells upon treatment with MERS-CoV spike polyclonal antibody, compared to the mock-treated group **(b)** and **(c)** Bar graph showing the percentage of neutralization of MERS-CoV PV infection in Huh7 cells and HEK293T cells transiently expressing DPP4, respectively **d** Relative percentage difference in MERS-CoV PV infection on HEK293T cells transiently expressing either pcDNA, DPP4 alone, TMPRSS2 alone or DPP4 + TMPRSS2 both **(e**–**g)** Percentage of relative MERS-CoV PV infection in Huh7, Vero and Calu3 respectively on treatment with 100 µM Camostat mesylate, *N* = 3, bar graphs represent mean ± SEM, ns non-significant, * *p* < 0.05; ** *p* < 0.01; *** p *<* 0.001; **** *p* < 0.0001.

We recently showed that SARS-CoVs and MERS-CoV pseudoviruses (CoV-PVs) enter cells by receptor-mediated endocytosis, which is demonstrated by significant inhibition of infection in cells treated with NH_4_Cl or Dynasore, which inhibit pH-dependent or dynamin-dependent endocytosis pathway, respectively. In order to conduct a more comprehensive study of the dual entry pathways utilized by MERS-CoV, which include direct membrane fusion and endocytosis, we generated VSV-based pseudotyped MERS-CoV (MERS-CoV PV) as described elsewhere^45^ and evaluated the permeability of MERS-CoV PV in both susceptible and non-susceptible cell lines. We found that MERS-CoV PV was permeable in both Vero and Huh7 cells but not in non-susceptible HEK293T cells. Furthermore, incubation of MERS-CoV PV with anti-MERS-CoV polyclonal antibodies neutralized the virus (Fig. 1a), indicating that the pseudotyped viruses presented a MERS-CoV spike on their surface and are permeable only in susceptible cells. Moreover, non-susceptible cells became permeable after transiently expressing DPP4 (Fig. 1b, c). These viruses were used for subsequent infection studies.

Next, we conducted experiments on HEK293T cells that lack both DPP4 and the membrane-bound serine protease TMPRSS2. We transiently expressed DPP4, TMPRSS2, or both in 293T cells and evaluated MERS-CoV pseudovirus infection. Our findings indicated that cells that were co-transfected with DPP4 and TMPRSS2 exhibited 4.4-fold higher levels of MERS-CoV pseudovirus infection than cells that were transfected with DPP4 alone. In contrast, cells that were transfected with TMPRSS2 alone did not show any infection (Fig. 1d). This suggests that MERS-CoV uses both entry pathways. We also studied entry pathways in human bronchial epithelial cells (Calu-3), human hepatoma cells (Huh7), and African green monkey kidney epithelial cells (Vero), which differentially express TMPRSS2. Treatment with Camostat, a drug that inhibits TMPRSS2 activity, considerably inhibited MERS-CoV PV infection in Calu-3 and Huh7 cells (Fig. 1e, f) that express endogenous TMPRSS2. Vero cells, which lack TMPRSS2, did not show any inhibition (Fig. 1g). These findings suggest that MERS-CoV enters cells through both endocytosis and direct membrane fusion. In contrast, cells such as HEK293T and Vero cells, which do not express endogenous TMPRSS2, rely only on the endocytic pathway for MERS-CoV entry.

### DPP4 cytoplasmic tail lacks a conserved endocytic motif

**Fig. 2:**
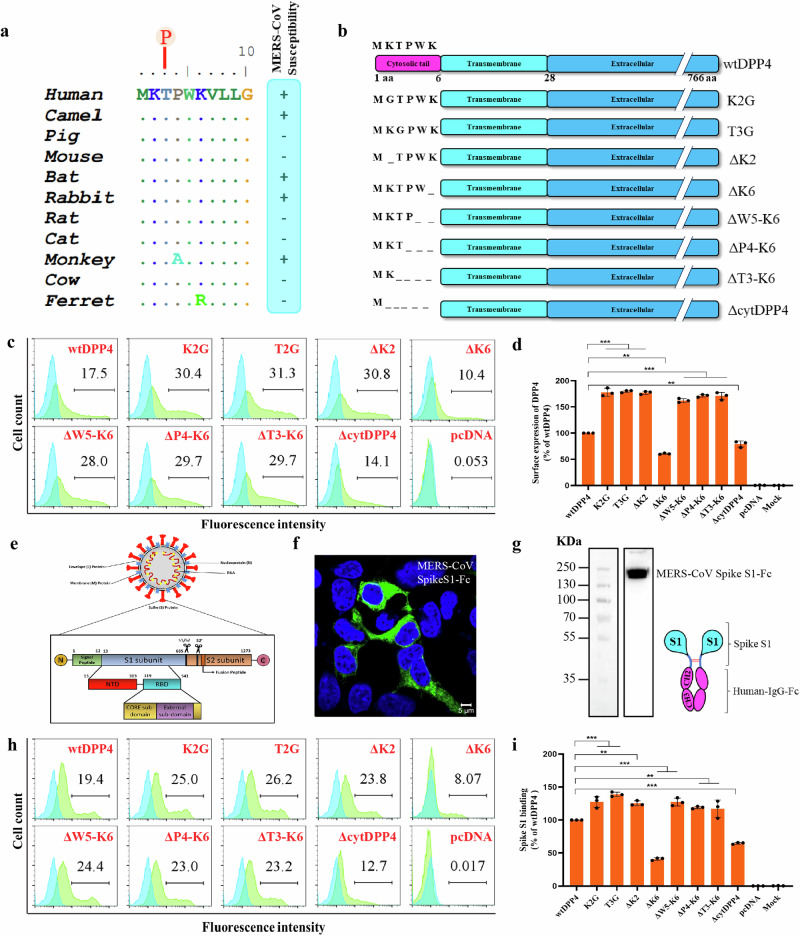
Surface expression and MERS-CoV S1-Fc binding of DPP4 mutants. **a** Multiple sequence alignment of DPP4 in different MERS-CoV susceptible and non-susceptible species, marking the putative phosphorylation site at the third amino acid position (letter ‘P’ marked in red). **b** Schematic representation of different mutations introduced in the cytoplasmic tail of DPP4. **c** Histograms depicting surface expression of wtDPP4 and its other mutants using flow cytometry. The values in the histogram depicts the percentage of FITC-positive cells. **d** Percentage difference of different DPP4 mutants surface expression as compared to wtDPP4. **e** Schematic representation of coronavirus spike protein. **f** In vitro expression confirmation of recombinant MERS-CoV spike S1-Fc protein using immunocytochemistry, scale bar = 5 µm **(g)** Molecular mass confirmation of purified recombinant MERS-CoV spike S1-Fc protein through western blot. **h** Histograms depicting surface binding of MERS-CoV spike S1 on DPP4 and its other mutant expressing cells using flow cytometry, values in the histogram plots indicate the percentage of FITC positive cells. **i** Percentage difference of MERS-CoV spike S1-Fc protein binding on different DPP4 mutants as compared to wild-type DPP4, *N* = 3, bar graph represents mean ± SD, ** *p* < 0.01,*** *p* < 0.001.

DPP4 is an aminopeptidase that is 766 aa in length and has an ectodomain of 738 aa, a transmembrane domain of 22 aa, and a short cytoplasmic tail of 6 aa. The cytoplasmic tail of DPP4 is relatively conserved among different MERS-CoV susceptible and non-susceptible species (Fig. 2a) and contains a putative phosphorylation site at amino acid position 3 (threonine, T3) (Fig. 2a). It is known that the cytoplasmic tail interacts with the cellular protein and activates NF-κB signaling during immune responses in immune cells, but it is not known whether this interaction occurs in other cell types and is involved in viral entry. Based on this, we hypothesize that the cytoplasmic tail of DPP4 might have a role in facilitating the internalization of MERS-CoV through mechanisms independent of the conserved endocytosis motifs. To better understand the role of the cytoplasmic tail of DPP4 in MERS-CoV entry, we introduced various substitution and deletion mutations in the human DPP4 cytoplasmic tail, such as point mutations at residues K2GDPP4 (K2G) and T3G DPP4 (T3G), deletion of single or multiple residues ΔK2DPP4 (ΔK2), ΔK6DPP4 (ΔK6), ΔW5-K6DPP4 (ΔW5-K6), ΔP4-K6DPP4 (ΔP4-K6), ΔT3-K6 DPP4 (ΔT3-K6), and a complete cytoplasmic tail deleted DPP4 (ΔcytDPP4) (Fig. 2b). Subsequently, the expression, surface presentation, and functionality of mutant DPP4 were analyzed by confocal imaging and in vitro functional studies.

### DPP4 mutants are expressed and localized to the cell membrane

In order to visualize the expression pattern and localization of DPP4 and its mutants, we transfected HEK293T cells with wtDPP4 and mutant DPP4 plasmids. The DPP4 was then stained using fluorescently labeled anti-DPP4 antibodies and analysed by flow cytometry. Upon transfection, all mutant constructs were expressed and inserted into the cell membrane. However, the constructs with ΔcytDPP4 and one of the single amino acids deleted, ΔK6DPP4, showed less number of surface expressing cells compared to wtDPP4 (Fig. 2c). A similar observation was noted in the fluorescent intensity analysis, where ΔcytDPP4 showed significantly less surface expression, with 12-14.4% of the transfected cells, while ΔK6DPP4 had ~10% of the cells positive for surface expression, which is much less than ΔcytDPP4 (Fig. 2d). In contrast, other mutants, such as K2G, T3G, ΔK2, ΔW5-K6, ΔP4-K6, and ΔT3-K6, showed elevated surface expression compared to wtDPP4 (Fig. 2d).

### Cytoplasmic tail deletion of DPP4 allows the binding of the MERS-CoV spike S1 protein

**Fig. 3:**
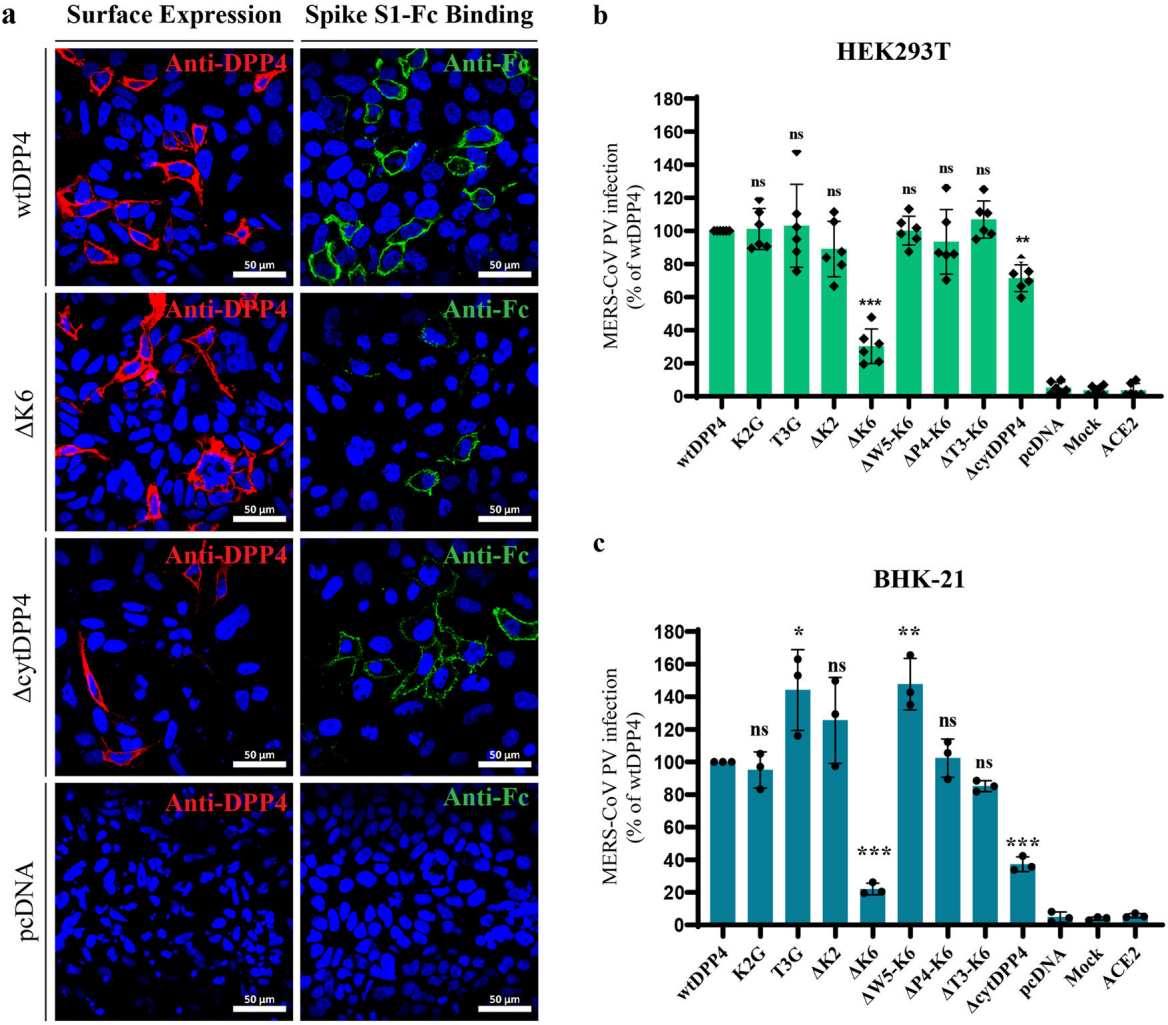
MERS-CoV spike interaction with mutant DPP4 and permissivity of MERS-CoV pseudotyped virus in DPP4 mutant expressing cells. **a** HEK293T cells transiently transfected with wtDPP4, ΔK6DPP4, ΔcytDPP4 or pcDNA showing receptor expression (red) and MERS-CoV spike S1 protein binding (green) upon immunostaining, scale bar = 50 µm (**b**) and (**c**) MERS-CoV infection in two non-susceptible cell lines HEK293T and BHK-21 respectively, transiently expressing wtDPP4 and its different mutants, *N* = 2, bar graphs represent mean ± SEM (HEK293T) and mean ± SD (BHK-21), * *p* < 0.05; ** *p* < 0.01; *** *p* < 0.001.

Proper cell surface presentation of membrane receptors is critical for the attachment of viruses and their entry into host cells^46^. The coronavirus spike consists of two subdomains, S1 and S2 (Fig. 2e). The S1 subdomain contains an N-terminal domain (NTD) and a receptor-binding domain (RBD), which are essential for interaction with the virus receptor^47,48^ while S2 facilitates virus-host membrane fusion^49^. Spike S1 domain of MERS-CoV binds to the ectodomain of DPP4 to facilitate viral entry^50,51^. To investigate whether the ΔcytDPP4 and other DPP4 mutants allow the binding of the MERS-CoV spike. We generated the recombinant S1 domain of the MERS-CoV spike, C-terminally fused with the Fc domain of human IgG (S1-Fc) (Fig. 2f, g), and produced it as a dimeric protein, in order to retain the integrity of the protein during purification steps and ensuring the protein functionality. Next, the recombinant protein was allowed to bind to the HEK293T cells expressing mutant DPP4, and the receptor-spike interaction was measured by flow cytometry analysis. We could see the interaction of S1-Fc on all the different DPP4 mutant-expressing cells, without any notable differences between these mutants but not on the empty plasmid transfected cells (Fig. 2h). However, the number of S1-bound cells was different between mutants. The ΔcytDPP4 showed a lower number of positive cells (12%), and the ΔK6DPP4 showed even less binding (~8%) compared to wtDPP4 (Fig. 2i). A similar pattern was observed when cells were transfected with increasing or decreasing the plasmid concentrations. Overall these data clearly show that the MERS-CoV spike could interact with all mutant DPP4, including ΔcytDPP4. But the reduction in MERS-CoV S1-Fc binding in ΔcytDPP4 and ΔK6DPP4 is consistent with the surface presentation of these receptors (Fig. 2c). Next, to check whether the observed signals are on the cell membrane, we performed confocal image analysis. We found that all the mutant DPP4 expressed on the cell surface and allowed binding of MERS-CoV S1-Fc, including ΔcytDPP4 and ΔK6DPP4 (Fig. 3a, Supplementary Fig S1). A clear surface presentation and S1 binding were observed only in DPP4 and mutants transfected cells, not in the controls. Notably, surface presentation and S1 binding were displayed by ΔcytDPP4 and ΔK6DPP4.

### Pseudotyped MERS-CoV is permeable in cells expressing mutant DPP4

Next, to investigate whether DPP4 mutants and ΔcytDPP4 can mediate MERS-CoV entry, ΔcytDPP4 and DPP4 mutants were transiently expressed in non-susceptible HEK293T and BHK-21 cells and then infected with MERS-CoV PV. Not with ACE2 and empty plasmid, but all mutant plasmid transfected cells were permeable to infection in both cell lines, with varying levels of infection between mutants (Fig. 3b, c). Notably, ΔcytDPP4 exhibited a 20–30% reduction in MERS-CoV PV infection in HEK293T cells and a more pronounced reduction of 60–70% in BHK-21 cells. Similarly, ΔK6DPP4 showed significantly less infection (60–80%) in both cell lines compared to wtDPP4 (Fig. 3b, c). The reduced infections were correlated with the surface expression and S1-Fc binding of the different mutant DPP4.

**Fig. 4:**
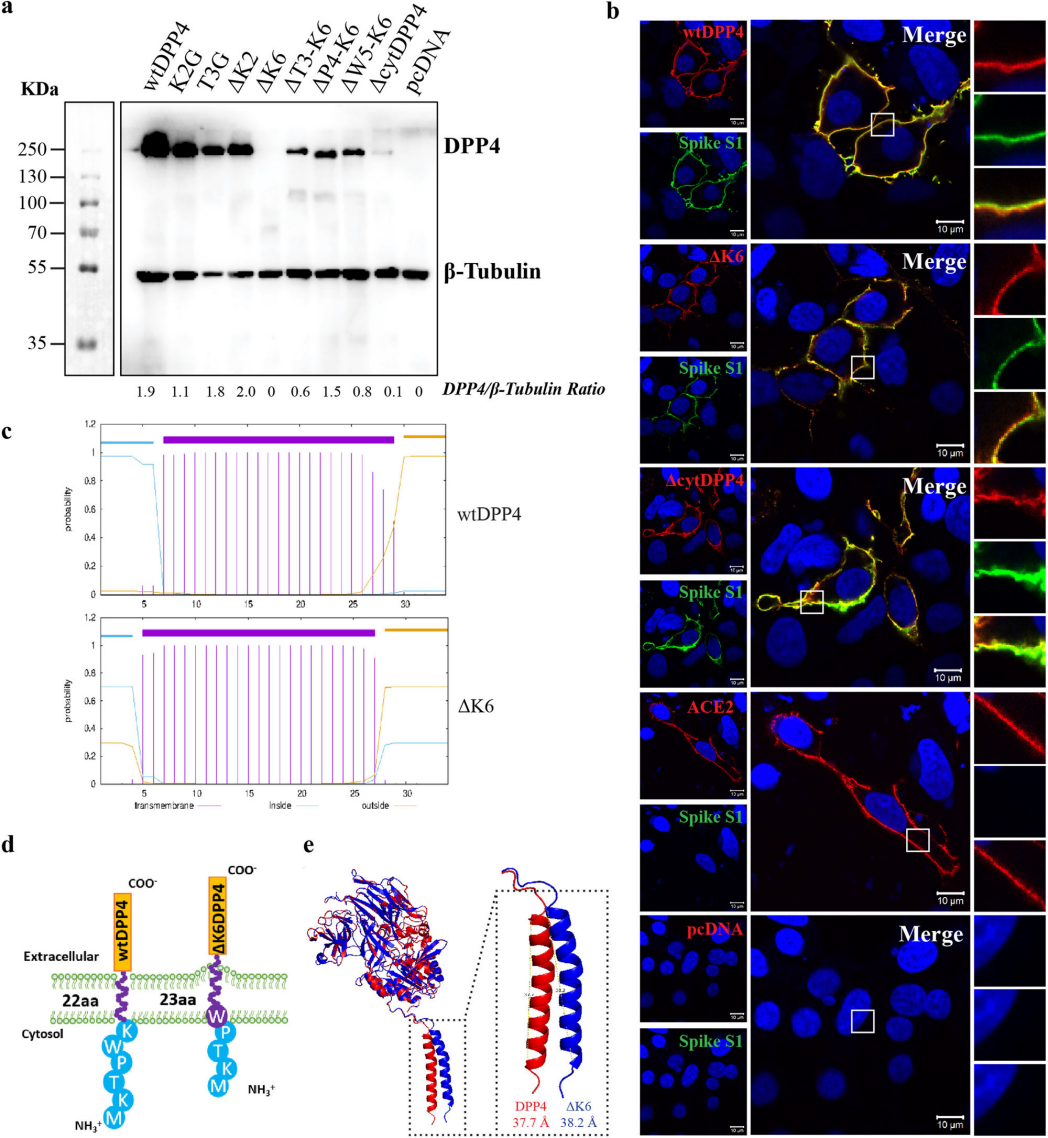
Protein expression and colocalization of DPP4 mutants. **a** Differential expression of wtDPP4 and its mutants (250 KDa) as analyzed by western blotting, tubulin stained as loading control (55 KDa). **b** Dual staining of wtDPP4, ΔK6DPP4, ΔcytDPP4, ACE2 or pcDNA (red) and MERS-CoV spike S1 protein (green) to visualize colocalization (yellow), white squares indicate zoomed area, scale bar = 10 µm. **c** Transmembrane length prediction of wtDPP4 and ΔK6 using the TMHMM v2.0 tool. **d** Schematic representation of the predicted arrangement of ΔK6DPP4 on the cell membrane in comparison with wtDPP4. **e** Alphafold3 predicted models of wtDPP4 and ΔK6DPP4 superimposed on each other showing a bend and increase in length of ΔK6DPP4 transmembrane domain as compared to wtDPP4.

To further evaluate these findings, we performed SDS-PAGE and Western blot analysis on the cell lysate of different DPP4 mutant transfected cells. The results showed comparable protein levels for most mutant constructs, except for ΔcytDPP4 and ΔK6DPP4 (Fig. 4a, Supplementary Fig S4). In the case of ΔcytDPP4, a faint band was observed, whereas no detectable band was found for ΔK6DPP4. This observation was consistent across multiple experiments. Next, we quantified the DPP4 band intensities relative to tubulin using Fiji (ImageJ) software and found that, apart from ∆K6, ∆T3-K6, ∆W5-K6, and ∆cytDPP4, the other ratios are roughly comparable (Fig. 4a). The observed differences in band intensities in the Western blot analysis are mainly due to the transfection efficiency for each mutant and the solubility of membrane-bound proteins. Further, to confirm the observed interaction is indeed on the receptor and not elsewhere in the cell, we performed a DPP4-spike colocalization assay. The spike protein was allowed to bind to the DPP4 mutant transfected cells. Consequently, cells were probed for DPP4 and S1-Fc using DPP4 polyclonal and spike-specific antibodies, respectively. Not with empty or ACE2 plasmid-transfected cells, but mutant plasmid-transfected cells showed precise spike interaction with the ectodomain of DPP4 (Fig. 4b, Supplementary Fig. S2, S3), which clearly shows that the spike colocalized with DPP4 and not elsewhere in the cells. A similar observation was noted in all DPP4 mutants, including the two less-expressing mutants, ΔK6DPP4 and ΔcytDPP4 (Fig. 4b, Supplementary Fig. S2, S3). These results clearly show that DPP4 and mutants are expressed on the cell membrane and allow the binding of the MERS-CoV spike protein. However, the less expression of ΔcytDPP4 and ΔK6DPP4 and the undetectability of the latter proteins might be due to the retention of the expressed protein within the membrane or changes in the rate of protein turnover. Particularly, in the case of ΔK6DPP4, this might be caused by the hydrophobic tryptophan residue (W) at the fifth position, where the deletion of the adjacent lysine residue (K) at the sixth position extends the length of the transmembrane domain from 22 to 23 amino acids (Fig. 4c). This extension, most likely hinders protein folding, surface presentation (Fig. 4d). We also modelled the structure of the ΔK6 mutant using the AlphaFold 3 server and compared it to the wtDPP4 and found that the transmembrane helix in ΔK6 is slightly longer, measuring 38.2 Å compared to 37.7 Å in wtDPP4. Furthermore, when **s**uperimposed on wtDPP4, ΔK6 shows a slight bend in the transmembrane region, which might affect the folding, stability, and surface presentation of the protein (Fig. 4e).

### MERS-CoV is permissive in cells that stably express the ΔcytDPP4 receptor

**Fig. 5:**
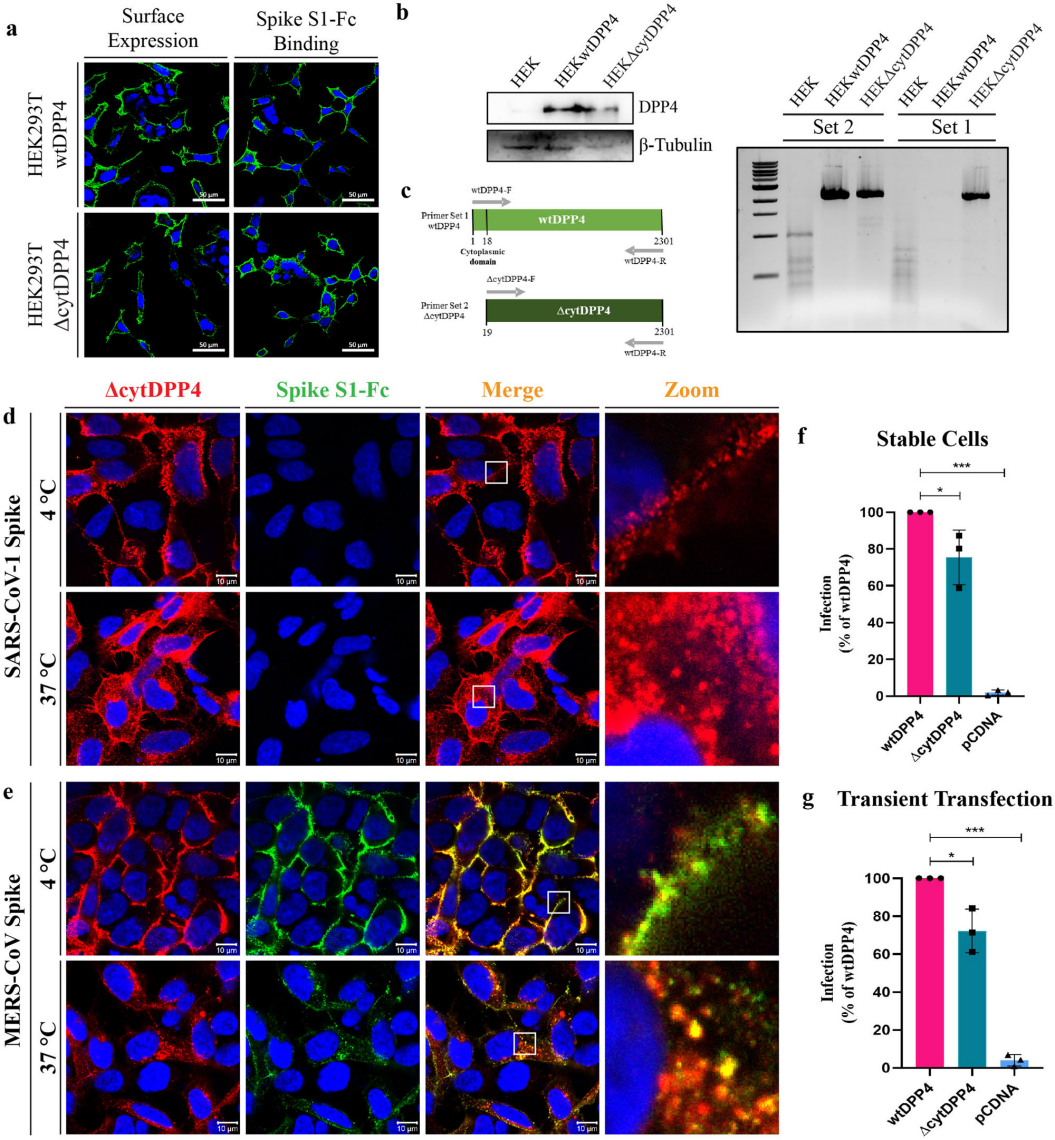
MERS-CoV pseudotyped viruses are permissive in cells stably expressing ΔcytDPP4. HEK293T stable cell lines expressing either full-length DPP4 ((HEK293TwtDPP4) or cytoplasmic tail-deleted DPP4 (HEK293TΔcytDPP4) were used to assess. **a** Cell surface expression and MERS-CoV S1 binding (scale bar = 50 µm), **b** Expression levels of wtDPP4 and ΔcytDPP4 by Western blot, and **(c)** confirmation of the cytoplasmic tail deletion in ΔcytDPP4 using reverse transcription PCR (RT-PCR) with two distinct primer sets, “Set1” primers were designed to amplify the both full-length DPP4 and ΔcytDPP4 sequence, while “Set2” primers include a forward primer binding site located immediately downstream of the cytoplasmic tail region, allowing amplification only of ΔcytDPP4. **d** Dual staining of ΔcytDPP4 (red) and SARS-CoV-2 spike S1 protein (green) to visualize colocalization (yellow), white square indicates zoomed area, scale bar = 10 µm. **e** Dual staining of ΔcytDPP4 (red) and MERS-CoV spike S1 protein (green) to visualize colocalization (yellow), white square indicates zoomed area, scale bar = 10 µm, **(f)** and **(g)** Percentage of relative pseudovirus infection in HEK293T^wtDPP4^ and HEK293T^ΔcytDPP4^ stable cell lines and cells transiently expressing wtDPP4 or ΔcytDPP4 respectively, *N* = 3, bar graphs represent mean ± SD, ns non-significant, * *p* < 0.05; ** *p* < 0.01; *** *p* < 0.001.

MERS-CoV PV was permeable in cells transiently transfected with ΔcytDPP4, but the infection level was significantly less than that of wtDPP4. To further confirm these findings, we generated HEK293T cells stably expressing either ΔcytDPP4 (HEK293T^ΔcytDPP4^) or wtDPP4 (HEK293T^wtDPP4^) (Fig. 5a, b). Both HEK293T^ΔcytDPP4^ and HEK293T^wtDPP4^ cells successfully transcribed their respective transcripts of DPP4 (Fig. 5c) and were expressed and localized on the cell membrane (Fig. 5a). The SARS-CoV-1 S1-Fc did not bind (Fig. 5d), whereas MERS-CoV S1-Fc showed binding to HEK293T^ΔcytDPP4^ and co-internalized into the host cell upon incubation at 37 °C (Fig. 5e). Subsequently, we investigated MERS-CoV PV infection in HEK293T^ΔcytDPP4^ and HEK293T^wtDPP4^ cells. Our results showed that both HEK293T^wtDPP4^ and HEK293T^ΔcytDPP4^ cells were permeable to MERS-CoV PV (Fig. 5g), and a similar observation was seen in transiently expressed cells (Fig. 5f). In contrast to the considerable variation seen in transiently expressed cells, we did see a slight reduction in infection in HEK293T^ΔcytDPP4^ cells compared to HEK293T^wtDPP4^ but did not find significant variations in infection levels between these two stable cell lines (Fig. 5d). These findings demonstrate that the absence of the cytoplasmic tail, DPP4 presented on the cell membrane, allows for the binding of the MERS-CoV spike protein and its subsequent internalization into host cells. Therefore, we speculate that MERS-CoV binds to DPP4, and following receptor binding, another host protein may trigger its internalization process.

## Discussion

The entry process of MERS-CoV is primarily mediated by the membrane-bound receptor DPP4, also known as CD26, which belongs to the family of peptidases^13^. DPP4 plays a key role in several physiological functions and is expressed in various tissues and organs, including the lungs, kidneys, intestine, liver, and immune cells^52^. DPP4 primarily cleaves peptides and hormones and binds to adenosine deaminase to activate T-cells^16,20^. In addition to its regular physiological function, DPP4 acts as the primary entry receptor for MERS-CoV^13^. The trimeric form of the MERS-CoV spike protein binds to DPP4 and facilitates viral entry into host cells. Similarly, other human coronaviruses (HCoVs) use peptidase family proteins as primary entry receptors. Notable examples include SARS-CoV-1, HCoV-NL63, and SARS-CoV-2, which use ACE2 as their primary receptor, and HCoV-229E, which uses aminopeptidase N (APN)^53^. Polyclonal antibodies against ACE2, APN, or DPP4 block the entry of the respective viruses SARS-CoV-1 and -2, HCoV-NL63, and MERS-CoV, respectively^54–56^. Additionally, receptor knockout cells are not susceptible to virus infection^57,58^. Therefore, it was believed that the primary receptor is solely responsible for the entry into host cells. Despite the primary receptors having a crucial role in entry, it is unclear how these viruses internalize into the host cell after binding to the receptors. Nevertheless, the involvement of cytoplasmic tails in facilitating viral internalization has been documented for receptors in other viral families. For example, the TIM-1 receptor for the dengue virus contains two lysine residues in its cytoplasmic domain that, upon ubiquitination, facilitate the virus endocytosis^59^. Similar to this, viral internalization is facilitated by the phosphorylation of the cytoplasmic tails of integrin during human cytomegalovirus (HCMV) entry^60^. Recently, we and others showed that the cytoplasmic domain of ACE2 is not critical for SARS-CoV-1 and -2 internalization^39,61^. However, the role of DPP4 cytoplasmic tail in virus entry is not clear yet.

The MERS-CoV receptor DPP4 contains a short 6-amino acid cytoplasmic tail, which is known to interact with the cellular protein CARMA1, a scaffold protein essential for T lymphocyte activation^62^. In this study, we showed that the DPP4 cytoplasmic tail is not crucial for MERS-CoV internalization. Our initial findings demonstrate that MERS-CoV can enter cells via direct membrane fusion and endocytosis. In comparison to DPP4 alone, the infection levels in cells co-transfected with DPP4 and TMPRSS2 are significantly higher, underscoring the critical role of TMPRSS2 in facilitating viral entry. Furthermore, the partial reduction of infection in cells endogenously expressing both DPP4 and TMPRSS2 following treatment with a TMPRSS2 inhibitor, in contrast to the lack of inhibition in cells expressing only DPP4, confirms the dual entry path of MERS-CoV. These findings are consistent with prior research^42^ and provide further insight into the mechanics of MERS-CoV entry. Next, we generated different combinations of deletion and substitution mutations in the cytoplasmic tail of DPP4 based on various regulatory functions of these residues such as putative ubiquitination and phosphorylation site. Moreover, we introduce mutations which enable more flexible confirmations and less steric hindrance within protein^63–66^ to effectively study the significance of the DPP4 cytoplasmic tail in viral entry. The mutant plasmids were independently analyzed for their expression and cell surface presentation. We found that all the mutant constructs were expressed in the cells and presented on the cell membrane. However, except for ΔK6 and ΔcytDPP4, all other mutations showed slightly higher expression of DPP4 on the cell surface. Protein studies have shown that single amino acid changes can increase expression and stability in a variety of proteins, including G protein-coupled receptors (GPCR) and green fluorescent protein (GFP)^67–69^. Mutations in DPP4, including K2G, T3G, ΔK2, ΔT3-K6, ΔP4-K6, and ΔW5-K6, might alter the stability or turnover rate of DPP4, resulting in increased surface expression. However, additional study is required to confirm this finding. The expression and presentation of ΔcytDPP4 was significantly less than wtDPP4, which is consistent with the cytoplasmic domain deleted ACE2, where membrane presentation was less than that of wtACE2^39^. It has been shown that the transmembrane domain of a protein is crucial for its proper integration into the membrane and correct folding. Deletion of the cytoplasmic domain of low-density lipoprotein receptor (LDLR), epidermal growth factor receptor (EGFR), and GPCRs disrupt their internalization and trafficking, leading to altered surface expression and receptor recycling^70–72^. We also noted that the expression and presentation of a single amino acid deleted ΔK6DPP4 was much less than that of ΔcytDPP4. This might be due to the deletion of the lysine (K) residue at the sixth position, which allowed the extension of the transmembrane domain slightly longer. The hydrophobic residue tryptophan (W) at the fifth position joins together with the 22 aa transmembrane domain, extending it to 23 aa, which might alter the protein synthesis, stability, and folding. Similarly, alterations, such as the addition of an amino acid, can disrupt the hydrophobicity and length of the transmembrane domain, potentially leading to misfolding or poor membrane presentation. Incorrectly folded proteins are often retained in the endoplasmic reticulum (ER) and are not transported to the cell surface^73–75^. Further, structural analysis showed that the transmembrane helix in ΔK6 is slightly extended and bent in the transmembrane region. Studies have demonstrated that a membrane protein structure and function can be significantly altered by a minor bending in the transmembrane region, which affects stability, folding, and ligand interactions. The helix packaging within the lipid bilayer can be disrupted by such bends, resulting in misfolding or altered trafficking^76^. The significance of precise helix orientation for membrane protein integrity has been proven by the fact that even minor alterations in transmembrane helices in receptors and channels can alter signaling or gating functions^77,78^. Subsequent functional studies are required to verify these structural predictions.

Proper membrane presentation of DPP4 is critical for efficient substrate binding and catalytic activity. In this study, we did not explicitly demonstrate the catalytic activity of DPP4, but showed the surface expression of DPP4 and mutants. All DPP4 mutants expressed and presented on the membrane allowed the binding of the MERS-CoV spike protein, consistent with previous studies^39^. Although two mutants, ΔcytDPP4 and ΔK6DPP4, showed reduced spike binding compared to wild-type DPP4, similar observations were seen in MERS-CoV pseudovirus infections. This suggests that DPP4 and mutant plasmids express on the cell membrane, allowing virus binding and entry. Interestingly, HEK293T cells stably expressing complete cytoplasmic tail deleted DPP4 still presented the protein on the cell membrane and allowed binding and entry of the MERS-CoV pseudovirus. Previous studies have shown that cytoplasmic domain deleted ACE2 can also be present on the membrane and facilitate the entry of SARS-CoV-1 and -2^39^. The DPP4 lacks a conserved endocytic motif in the cytoplasmic tail. In the absence of a cytoplasmic tail, the MERS-CoV spike can bind and internalize to the cells, suggesting that the cytoplasmic tail of DPP4 is not critical for MERS-CoV entry, and this phenomenon might extend to other viral receptors in the peptidase family.

We speculate that the DPP4 might act as the primary receptor, but the entry might be mediated by other cellular factors or co-receptors, as seen in other well-studied enveloped viruses such as hepatitis C virus (HCV) and human immunodeficiency virus (HIV). Both HIV and HCV entry is a complex multistep process, HIV first attaches to the primary receptor CD4 on the host cell surface, followed by interaction with co-receptors CCR5 or CXCR4, which is required for viral entry^34,79^. These interactions mediate the fusion of the viral membrane with the host cell membrane. The cytoplasmic tail of the CCR5 contains a dileucine motif critical for its internalization and the subsequent virus entry into T cells^80^. Similarly, HCV also uses a complex multi-step process to enter host cells, involving several host cell proteins and receptors, including the scavenger receptor, claudin-1, occludin, and tetraspanin CD81. These host factors work together to facilitate HCV entry into host cells^79,81–83^. In contrast, influenza virus entry is facilitated by the cytoplasmic domain of the primary receptor, which contributes to the endocytic uptake of the virus into the host cells^84^. Recently, we have shown that the cytoplasmic domain of ACE2 signaling is not necessary for the internalization of both SARS-CoV-1 and -2^39^. The spike protein binds to the ectodomain of ACE2, and subsequent internalization might be facilitated by additional host factors to support viral entry^39^.

In conclusion, our study shows that the cytoplasmic tail signaling of DPP4 is not critical for the entry of MERS-CoV in HEK293T cells. MERS-CoV uses DPP4 as the primary receptor, and upon binding to the receptor, the virus or DPP4 may bind to other host factor(s) to initiate the internalization process like HCV and HIV. Further studies should focus on identifying these additional host factor(s) involved in the internalization process, which might provide insights into novel therapeutic targets for MERS-CoV and other coronaviruses.

## Methods

### Cell line maintenance

Human embryonic kidney 293T cells (HEK293T) were maintained in Dulbecco’s modified Eagle medium (DMEM, Lonza, 12604 F). Baby hamster kidney cells 21 (BHK-21, ATCC, ATCC- CCL-10) were maintained in Eagle’s minimal essential medium (EMEM, Lonza, 12-611 F). The human hepatoma cell line (Huh7) obtained from the Erasmus Medical Centre was maintained in RPMI-1640 medium (Lonza, 12115 F). Media was supplemented with 10% Fetal bovine serum (FBS, MP Biomedicals, 29101) and 1% penicillin/streptomycin antibiotic (Lonza, 17-602E) and the cells were grown at 37 °C in 5% CO_2_.

### Bioinformatics analysis of DPP4 sequences

Amino acid sequences of DPP4 from different species were retrieved from the Uniprot database (accession numbers: Human: P27487, Camel: W8G4M1, Pig: P22411, Mouse: P28843, Bat: M1PFC6, Rabbit: G1T1C1, Rat: P14740, Cat: Q9N2I7, Monkey: P12545, Cow: P81425, Ferret: M3XN99) and subjected to multiple sequence alignment using BioEdit software. The putative phosphorylation sites in the DPP4 cytoplasmic tail were predicted using NetPhos v3.1 software (https://services.healthtech.dtu.dk/services/NetPhos-3.1/). The length of the transmembrane domain and amino acid sequence information of DPP4 mutants were predicted using TMHMM v2.0 software (https://services.healthtech.dtu.dk/services/TMHMM-2.0/).

### Generation of plasmid constructs

The full-length human DPP4 (766 aa) gene was amplified from the cDNA library of Huh7 cells and cloned into eukaryotic expression plasmid, pcDNA3.1(+) using restriction-ligation cloning (designated as pcDNA-wtDPP4). To generate eight distinct constructs incorporating mutations in the cytoplasmic tail of the DPP4 gene, site directed mutagenesis was performed. The desired changes include two substitution mutations (K2G, T3G) and six independent deletion mutations (ΔK2, ΔK6, ΔW5-K6, ΔP4-K6, ΔT3-K6, ΔcytDPP4) in the cytoplasmic tail. The mutations were incorporated in the forward primers along with the restriction sites flanking at the 5’-end for subsequent cloning (Supplementary Table 1). Further, the wtDPP4 gene was PCR amplified from the pcDNA-wtDPP4 plasmid using the mutagenesis forward primer and the reverse primer in a reaction mix containing PfuUltra II Fusion High-fidelity Polymerase (Agilent,600672) following the manufacturer’s protocol. The amplified product was purified using FavorPrep GEL/PCR Purification Mini Kit (Favorgen Biotech Corp; FAGCK001-1) and cloned into pcDNA3.1(+) plasmid. All the nucleotide changes in the mutant constructs were validated using Sanger sequencing.

The full-length spike genes of MERS-CoV (GenBank: JX869059, 1321aa) and SARS-CoV-1 (GenBank: AY278491, 1236aa) were PCR amplified from the cDNA and the amplicons were cloned into pCAGGs expression vector (pCAGGS-MERS-CoV-S and pCAGGS-SARS CoV-1-S) as described elsewhere^39^. The spike S1-fc domain of both MERS CoV and SARS CoV-1 were selectively amplified from the full-length spike constructs, C-terminally tagged with human IgG-fc to produce dimeric soluble proteins and cloned into pCAGGs expression vector.

### Production of recombinant proteins

HEK293T cells were seeded in five 100 mm cell-culture dishes (ThermoFisher, 150466). At 70% confluency, either pCAGGS-MERS-CoV-S1-Fc or pCAGGS-SARS-CoV-1-S1-Fc plasmid was transfected using polyethylenimine (PEI, Polysciences, 23966) at a ratio 3:1 (PEI: DNA)^45^. Four hours post-transfection, the spent 1% DMEM media was replaced with FreeStyle expression media (Invitrogen, 12338018) supplemented with 1% penicillin/streptomycin and non-essential amino acids (NEAA, Lonza, BE13-114E). Five days following the media change, the supernatant was collected, and cell debris was removed by centrifugation at 1200 rpm for 10 min. The cleared supernatant was transferred into a new 50 mL conical centrifuge tube and then incubated with protein A Sepharose beads (Cytiva, GE17-0780-01) for 12 h at 4 °C with constant rotation. Following incubation, the supernatant was gently aspirated out, and the beads were collected in a 1.5 mL microcentrifuge tube and washed thrice using 1X PBS. All elution was done in tubes using 0.5 M glacial acetic acid, pH 3 (300 μL each). Elutes were neutralized using 3 M tris-Cl, pH 8.8 (150 μL). The quality and quantity of the protein were measured by spectrophotometer (Denovix, USA), SDS-PAGE, and Western blot analysis.

### Production of pseudotyped MERS-CoV (MERS-CoV PV)

We generated pseudotyped viruses of MERS-CoV as described elsewhere^51^. Briefly, to produce vesicular stomatitis virus (VSV) based pseudotyped MERS-CoV, spike S1 protein was transiently expressed in HEK293T cells. After 24 h of transfection, cells were infected with 1 MOI of VSVΔG/GFP virus for 1 h at 37 °C. Further cells were washed twice using 1X PBS and replaced with fresh DMEM containing 1% FBS and incubated for 24 h at 37 °C. Pseudotyped viruses were harvested and titrated in Huh7 cells. Pseudotyped MERS-CoV was validated for the receptor-specific entry. HEK293T cells were transfected with either DPP4 or pcDNA3.1(+) plasmids. After 24 h of transfection, 1 MOI of MERS-CoV PV was added to the transfected cells and incubated for 1 h at 37 °C to allow virus entry. Following the incubation, the fresh 1% FBS containing DMEM media was added to cells.

### Pseudovirus neutralization assay

The spike presentation of the MERS-CoV PV was tested by neutralization assay using spike specific polyclonal antibody. Monolayer of Huh7 cells were grown in a 96 well plate. MERS-CoV PV was incubated with spike polyclonal antibody at 1: 100 dilution for 1 h and the virus-antibody mix was added to Huh7 cells for infection. Twenty-four hours post-infection, the cells were analyzed under fluorescence microscope and the GFP-positive cells were counted.

### Analysis of DPP4 surface expression by immunofluorescence and flow cytometry

HEK293T cells were grown on coverslips at 70% confluency and transfected with 2 µg of expression plasmids encoding either wtDPP4, mutant DPP4, or pcDNA3.1 independently. Four hours post-transfection, the medium was replaced with DMEM containing 1% FBS, and the cells were maintained at 37 °C for 24 h. Coverslips were then washed twice with 1X PBS, and the cells were fixed with 4% paraformaldehyde (Sigma-Aldrich, P6148). Next, the cells were washed twice with 1X PBS and blocked with 1% bovine serum albumin (BSA, Sigma, A4503) for 1 h. Immunostaining was subsequently performed using a goat anti-DPP4 polyclonal antibody (1:300) for 1 h, followed by incubation with an Alexa Fluor 488-conjugated donkey anti-goat secondary antibody (1:500). Nuclei were stained using 4′,6-diamidino-2-phenylindole (DAPI). Images were captured using laser confocal microscopy (Zeiss LSM 880, Germany).

To perform flow cytometry, transfected cells were trypsinized and resuspended in ice-cold 1X PBS buffer. The cells were incubated with goat anti-DPP4 polyclonal antibody (1:250) for 40 min at 4 °C. After incubation, the cells were washed with ice-cold 1X PBS and then incubated with rabbit anti-goat antibody conjugated to Alexa Fluor 488 (1:500) for 30 min at 4 °C. The fluorescently labeled cells were analyzed using flow cytometry (BD FACS Aria III, USA). The percentage of DPP4-expressing cells was calculated by counting the number of FITC-positive cells, with the expression of wtDPP4 set at 100% for normalization. The FACS data was analyzed using FlowJo v10.8.1 software.

### MERS-CoV spike S1 binding and colocalization assay on mutant DPP4 receptors

HEK293T cells were grown on coverslips, and at 70% confluency, the cells were transfected with plasmids encoding wtDPP4 and DPP4 mutants using PEI. At 24 h post-transfection, the cells were treated with 5 μg/mL MERS-CoV spike S1-Fc on ice for 40 min. After incubation, the cells were washed with ice-cold 1X PBS. Receptor-bound spike S1 proteins were then detected with an anti-human IgG FITC-conjugated antibody (1:500) and incubated on ice for 40 min. The cells were then fixed using 4% PFA, and the nuclei were stained with DAPI. The coverslips were subsequently mounted on glass slides. Spike S1 binding was analyzed and quantified by confocal microscopy and flow cytometry, respectively. The binding of MERS-S1-Fc to cells expressing DPP4 mutants was calculated relative to wtDPP4.

To examine the colocalization of spike S1-Fc with wtDPP4 and mutant DPP4, we followed the procedure mentioned above, with the exception of antibody staining. The cells were fixed, and spike S1 proteins were stained with an anti-human IgG FITC-conjugated antibody for 1 h at 37 °C. DPP4 and ACE2 proteins were stained with a goat anti-DPP4 antibody and a goat anti-ACE2 antibody (1:300) for 1 h at 37 °C. Secondary antibody staining was performed using a rabbit anti-goat antibody conjugated with Alexa Fluor 594 (1:500). Nuclei were stained with DAPI.

### Generation of stable cell lines using the lentivirus transduction method

To generate HEK293T cells stably expressing wtDPP4 or ΔcytDPP4, we used a lentivirus-based system to integrate the gene into the cells. We cloned the full-length wtDPP4 or ΔcytDPP4 into the lentivirus transfer plasmid pQCXIN, which contains a neomycin selection marker. Next, we produced lentiviruses by co-transfecting pVSV-G (envelope plasmid), pBS-gag-pol (packaging plasmid), and pQCXIN-wtDPP4/ΔcytDPP4 (transfer plasmid) into HEK293T cells. The cell supernatant containing lentiviruses was harvested after 24 h and stored at -80 °C until further use. To produce stable wtDPP4 or ΔcytDPP4 HEK293T cells, the respective lentiviruses were used to infect the cells. Positive clones were then selected using neomycin antibiotic selection. HEK293T wtDPP4/ΔcytDPP4 cells were sorted using flow cytometry and further characterized. To validate the HEK293T wtDPP4/ΔcytDPP4 cell lines, Western blotting, reverse transcript analysis, and MERS-CoV spike S1 binding assays were performed.

To examine the expression of wtDPP4 and ΔcytDPP4, stable cells were grown in a 6-well dish and lysed using RIPA buffer for 30 min at 4 °C in the presence of protease inhibitors (Roche, 04693132001). Total protein concentration was measured using the BCA assay (Bicinchoninic acid assay, Pierce BCA assay kit, Thermo Scientific, 23227). Twenty micrograms of total protein from each sample were mixed with 5X SDS loading dye and incubated for 30 min at 37 °C. Samples were loaded into a 10% SDS-PAGE gel, and Western blotting was performed using a goat anti-DPP4 primary antibody (R&D, AF1180, 1:1000) and secondary with a rabbit anti-goat HRP-conjugated antibody (Immunotag, ITSAH238, 1:5000).

Total RNA was isolated from HEK293T^wtDPP4^ and HEK293T^ΔcytDPP4^ using a Qiagen tissue RNA isolation kit (Qiagen, 74104) and reverse transcription PCR (RT-PCR) was performed according to the manufacturer’s instructions. Briefly, complementary DNA (cDNA) was synthesized using SuperScript IV reverse transcriptase (ThermoFisher Scientific, 18090050), and two sets of oligos were used (primer set 1: stable-wtDPP4-F, stable-wtDPP4-R and primer set 2: stable-ΔcytDPP4-F, stable-ΔcytDPP4-R) to amplify full-length and cytoplasmic tail deleted DPP4, according to the protocol described elsewhere^39^.

### MERS-CoV spike S1-Fc internalization assay in wtDPP4 and ΔcytDPP4 stable HEK293T cells

To investigate whether HEK293T^ΔcytDPP4^ stable cells can mediate the internalization of MERS-CoV spike S1-hFc protein. Monolayer of HEK293T^ΔcytDPP4^ were grown on coverslips, and the cells were incubated with MERS-CoV or SARS-CoV-1 spike S1-hFc proteins (5 µg/mL) for 4 °C and 37 °C. Next, cells were washed with 1X PBS and fixed with 4% PFA for 10 min at room temperature (RT). Then the cells were permeabilized using 0.1% Triton X-100 for 5 min at RT and washed with 1X PBS. The binding of spike S1 proteins was detected using an anti-human IgG FITC conjugated antibody (Bethyl, A80119F, 1:500) for 1 h at 37 °C. To detect the DPP4 receptor, goat anti-DPP4 polyclonal antibody (1:300) was added and incubated for 1 h at 37 °C. Nuclei were stained with DAPI.

### MERS-CoV PV infection

HEK293T and/or BHK-21 cells were transiently transfected with either wtDPP4, DPP4 mutants, TMPRSS2, wtDPP4 + TMPRSS2 or pcDNA. HEK293T^wtDPP4/ΔcytDPP4^ were grown in a 96-well plate. Twenty-four hours post-transfection, cells were infected with MERS-CoV PV for 1 h at 37 °C. After 24 h infection, GFP-positive cells were counted and the relative percentage of infection was calculated. Mock in each condition refers to untreated/non-transfected cells.

### TMPRSS inhibition Assay

Huh7, Vero and Calu3 cells seeded in a 96-well plate were allowed to grow for 24 h at 37 °C. After attaining confluence, cells were treated with 100 µM Camostat mesylate (Sigma, SML0057) for 2 h at 37 °C. Cells were then infected with either MERS-CoV PV alone (control) or MERS-CoV PV supplemented with 100 µm Camostat mesylate (treated) for 1 h at 37 °C. Twenty-four hours post infection, GPF-positive cells were counted and calculated the relative percentage of inhibition.

### Modelling of wtDPP4 and ∆K6 mutant structure using Alphafold3

Full-length amino-acid sequence of human DPP4 (wtDPP4) was obtained from UniProt (ID: P27487). To construct the ∆K6 mutant, K (Lysine residue) at the 6th position was removed from the wtDPP4 sequence. Both the wtDPP4 and ∆K6DPP4 sequences were submitted separately to AlphaFold3 server (https://alphafoldserver.com/), which yielded five models for each sequence. Predicted model having the highest confidence score for each structure was picked for comparison. Both structures were opened in PyMOL2 and superimposed on each other using the ‘super’ command. The length between the two amino acids was measured using ‘measurement’ tool under the ‘wizard’ tab. The numerical in the image indicates value in angstrom (Å).

### Statistical analysis

The data presented in the manuscript are expressed as mean ± SD and were analyzed using GraphPad Prism software version 9. Statistical significance between groups was assessed using one-way ANOVA and Unpaired t test. Significance levels are indicated as follows: ns non-significant; * *p* < 0.05; ** *p* < 0.01; *** *p* < 0.001; **** *p* < 0.0001

## Supplementary information

Supplementary data

## Data Availability

No datasets were generated or analysed during the current study.
