# Supplementary material for: Middle East respiratory syndrome coronavirus (MERS-CoV) internalization does not rely on DPP4 cytoplasmic tail signaling
Source: Npj Viruses. 2024 Dec 30;2:67. doi: 10.1038/s44298-024-00080-y (PMC11721135; doi:10.1038/s44298-024-00080-y)
Supplement: Supplementary file 1 — Supplementary data [file 44298_2024_80_MOESM1_ESM.docx]

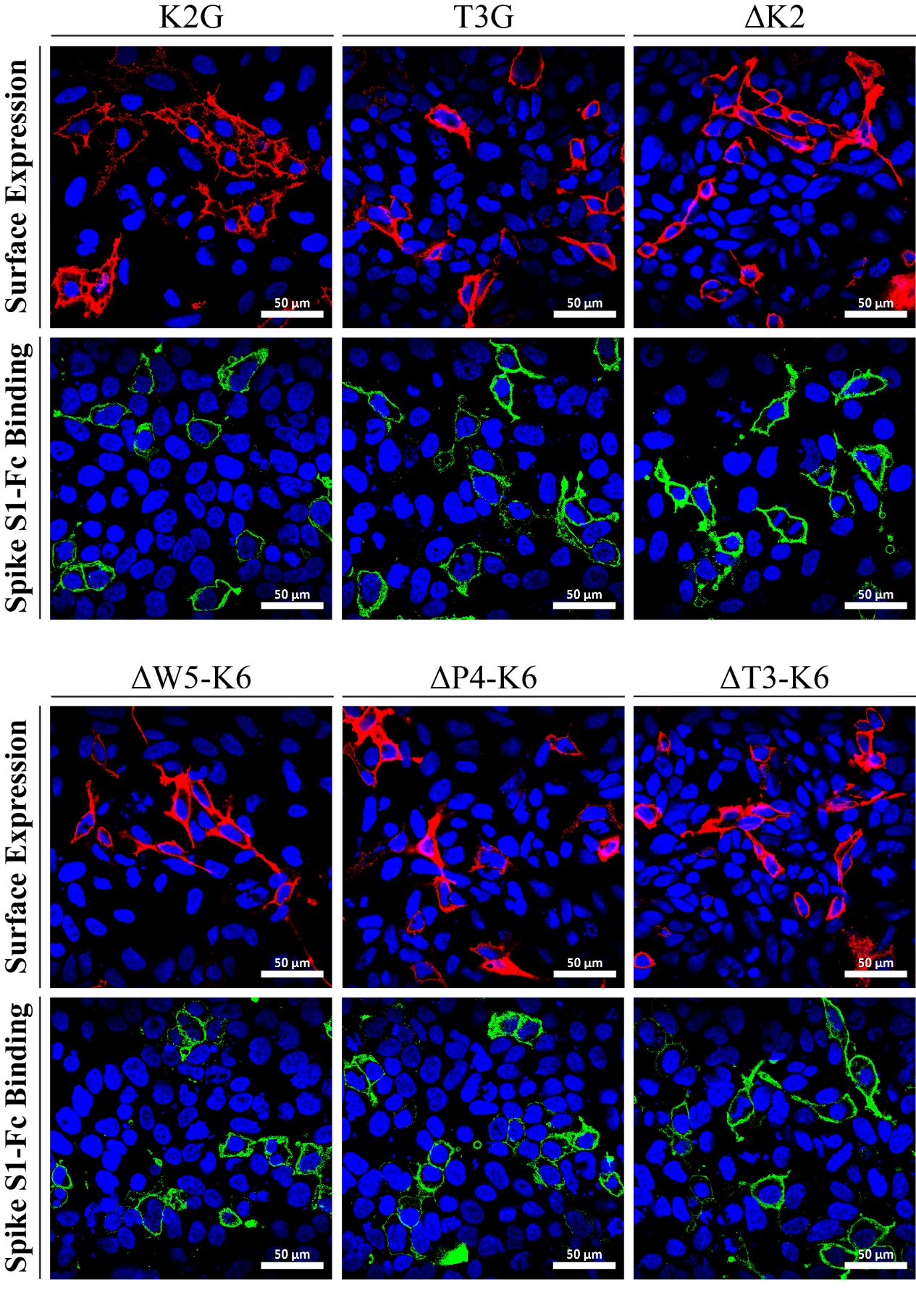


**Fig. S1: Receptor expression of DPP4 mutants and MERS-CoV spike S1 binding assay.** HEK293T cells transiently transfected with K2GDPP4, T3GDPP4, ΔK2, ΔW5-K6, ΔP4-K6 or ΔT3-K6 showing receptor expression (red) and MERS-CoV spike S1 protein binding (green) upon immunostaining, scale bar = 50 µm.


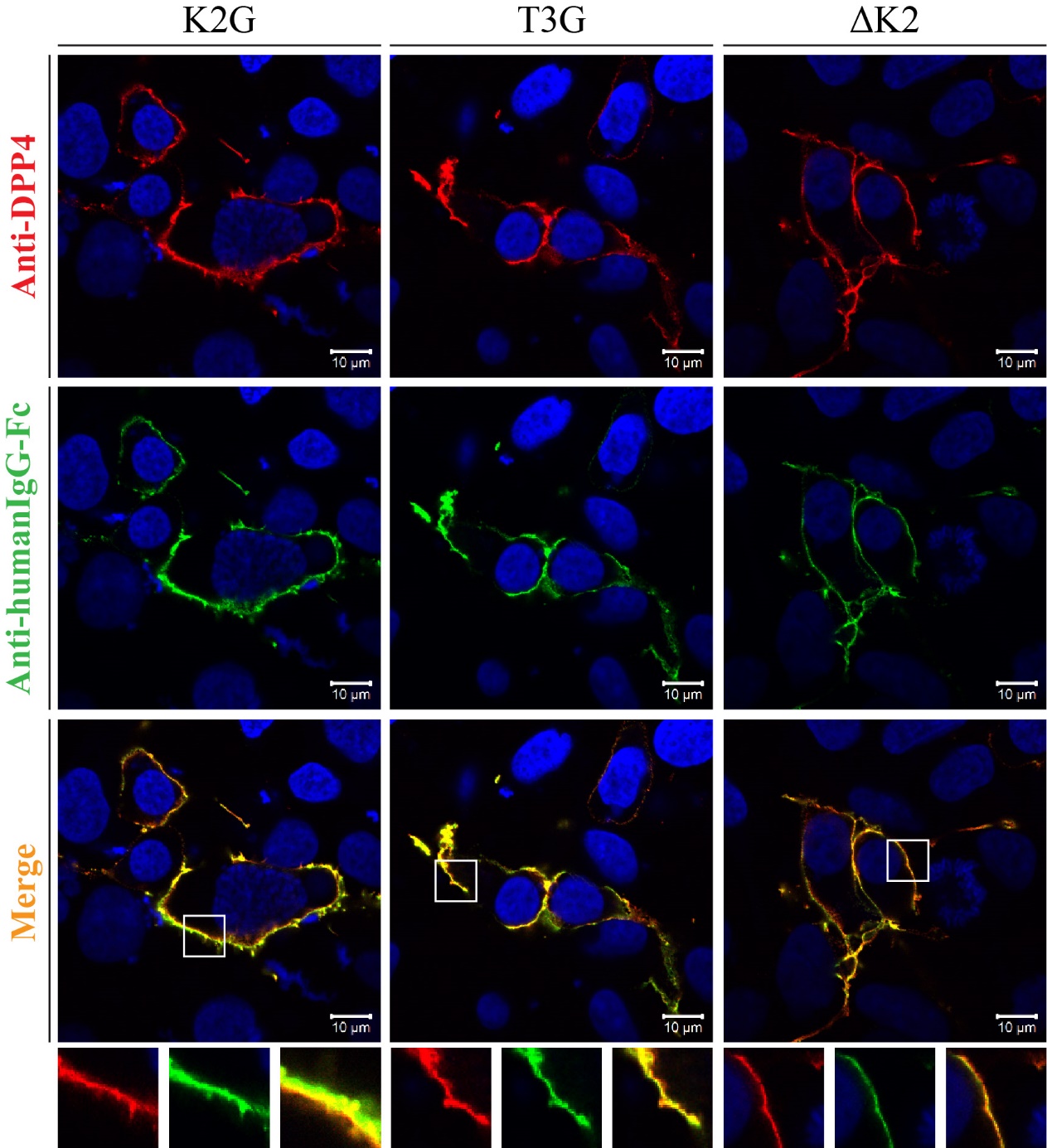


**Fig. S2: Colocalization of MERS-CoV spike S1 with DPP4 mutants.** Dual staining of K2GDPP4, T3GDPP4 or ΔK2 (red) and MERS-CoV spike S1 protein (green) to visualize colocalization (yellow) between the two. White squares indicate zoomed area, scale bar = 10 µm.


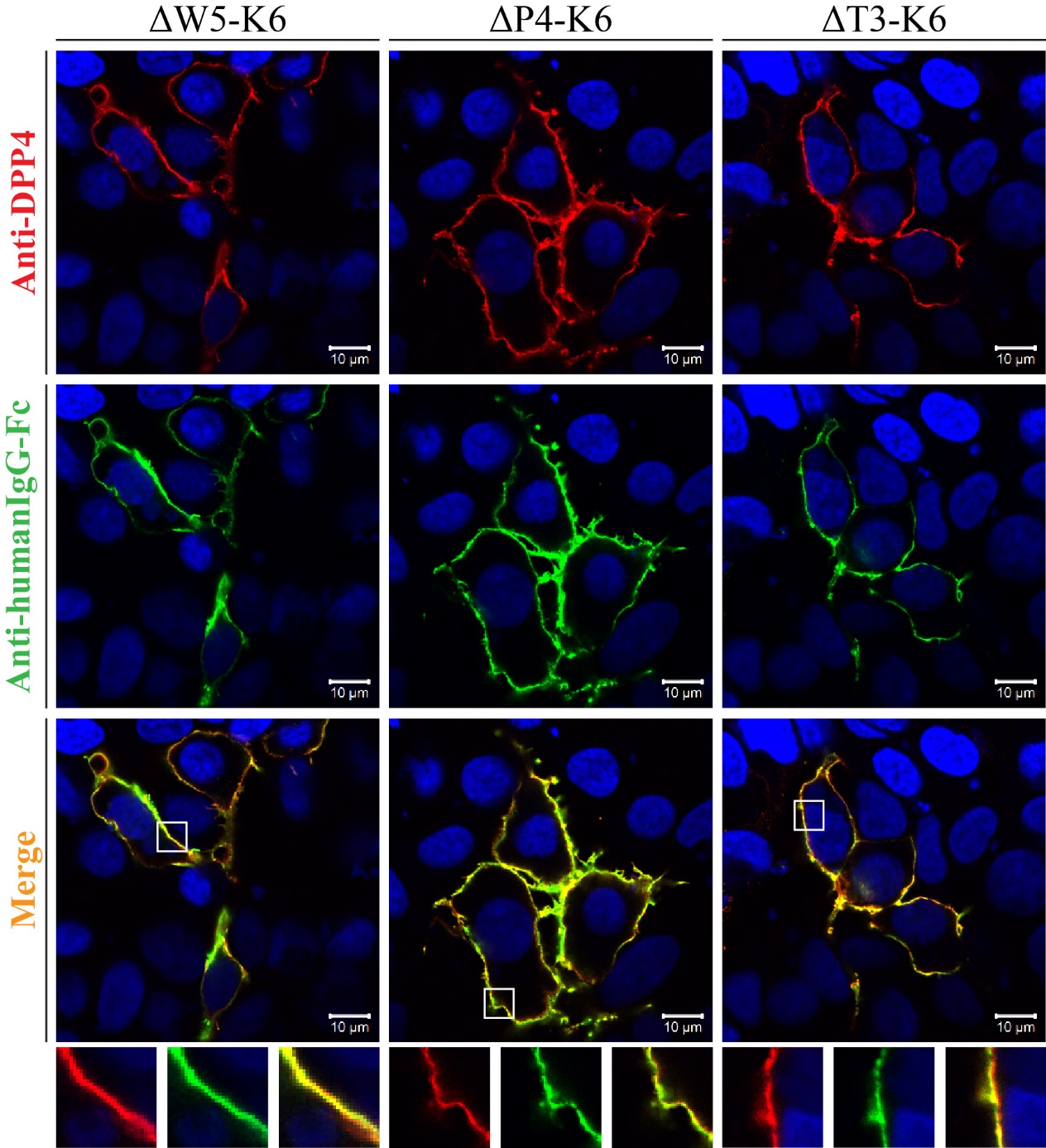


**Fig. S3:** **Colocalization of MERS-CoV spike S1 with DPP4 mutants.** Dual staining of ΔW5-K6, ΔP4-K6 or ΔT3-K6 (red) and MERS-CoV spike S1 protein (green) to visualize colocalization (yellow) between the two. White squares indicate zoomed area, scale bar = 10 µm.

**K2G**

**T3G**

**ΔK2**

**ΔK6**

**ΔT3-K6**

**ΔP4-K6**

**ΔW5-K6**

**ΔCytDPP4**

**WtDPP4**

**pCDNA**

**K2G**

**T3G**

**ΔK2**

**ΔK6**

**ΔT3-K6**

**ΔP4-K6**

**ΔW5-K6**

**ΔCytDPP4**

**WtDPP4**

**pCDNA**

c)

b)

a)


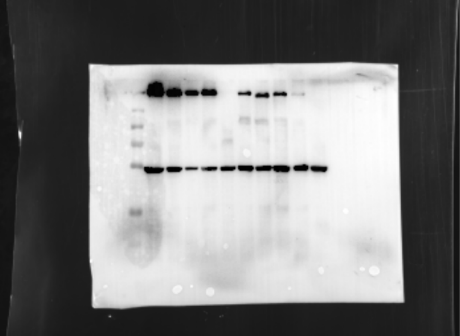

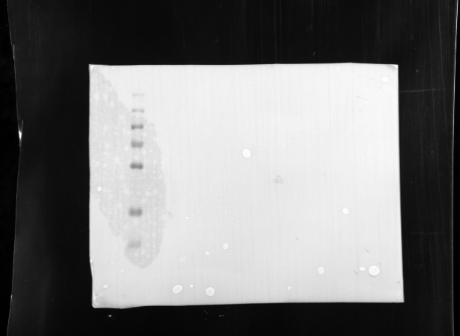

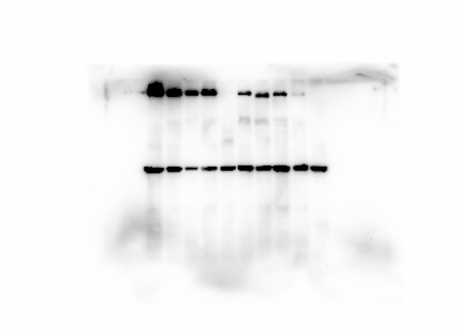


KDa

KDa

250

130

100

70

55

35

25

250

130

100

70

55

35

25

**Fig. S4:** Raw images of western blot showing wtDPP4 and its mutants **(a) (b)** and **(c)** Protein ladder, chemiluminescence and merged image respectively corresponding to the western blot in main figure panel (Fig. 4a). All images were captured using Bio-Rad ChemiDoc XRS+ system; Ladder images captured through Epi/White light whereas DPP4 bands were developed in the chemiluminescent mode; Ladder corresponds to PageRuler Prestained Protein Ladder: 10-250 KDa (Thermofisher). No adjustments in terms of cropping or any other non-linear adjustments were made to the image.

**Table S1: List of primers used to generate recombinant constructs in this study.**

| **Gene Description** | **Primer Code** | **Primer Sequence (5' - 3')** |
| --- | --- | --- |
| wtDPP4 RT-PCR | wtDDP4-RT-F | TATACCGGTGCCACCATGAAGACACCGTGGAAGG |
|  | wtDDP4-RT-R | TATTTAATTAACTAAGGTAAAGAGAAACATTG |
| ΔcytDPP4 RT-PCR | ΔcytDPP4-RT-F | TATACCGGTGCCACCATGGTTCTTCTGGGACTG |
| ACE2 | wtACE2-F | CTTGGTACCGCCACCATGTCAGGCTCTTYCTGG |
|  | wtACE2-R | CCGCTCGAGCTAAAABGAVGTCTGAACATCATC |
| wtDPP4 | wtDPP4-F | TATCTCGAGCATGAAGACACCGTGGAAGGT |
|  | wtDPP4-F | GCGGGATCCAGGTAAAGAGAAACATTGTT |
| ΔcytDPP4 | ΔcytDPP4-F | TATGGATCCACCATGGTTCTTCTGGGACTGCTGGG |
|  | ΔcytDPP4-R | GCGGGATCCAGGTAAAGAGAAACATTGTT |
| K2G | SDM-K2G-F | GGATCCACCATGAAGACACCGTGGGGGGTTCTTCTGGGACTGCTGGGTGC |
| T3G | SDM-T3G-F | TATGGATCCACCATGAAGGGACCGTGGAAGGTTCT |
| ΔK2 | K2Del-F | TATGGATCCACCATGACACCGTGGAAGGTTCTT |
| ΔK6 | K6Del-F | TATGGATCCACCATGAAGACACCGTGGGTTCTTCTGGGACTG |
| ΔW5-K6 | W5_K6Del-F | TATGGATCCACCATGAAGACACCGGTTCTTCTGGGACTGCTG |
| ∆P4-K6 | P4_K6Del-F | TATGGATCCACCATGAAGACAGTTCTTCTGGGACTGCT |
| ΔT3-K6 | T3_K6Del-F | TATGGATCCACCATGAAGGTTCTTCTGGGACTGCTGGG |
| DPP4 Stable Cells | stable-wtDPP4-F | TATACCGGTGCCACCATGAAGACACCGTGGAAGG |
|  | stable-wtDPP4-R | TATTTAATTAACTAAGGTAAAGAGAAACATTG |
| ΔcytDPP4 Stable Cells | stable-ΔcytDPP4-F | TATACCGGTGCCACCATGGTTCTTCTGGGACTG |
|  | stable-wtDPP4-R | TATTTAATTAACTAAGGTAAAGAGAAACATTG |
| SARS-CoV-1 Spike S1 Fc | SARS-1-S1Fc-F | TATGAGCTCAGTGACCTTGACCGGTGC |
|  | SARS--S1Fc-R | TATGGTACCCTAGTGGTGATGATGGTGGTGGCCTCGAGGAGCCACAATAGATT |

**Table S2: List of commercial antibodies used in this study**

| **S.No.** | **Antibody Name** | **Host** | **Company** | **Catalogue Number** |
| --- | --- | --- | --- | --- |
| 1 | Human DPPIV/CD26 Antibody | Goat | R&D Systems | AF1180 |
| 2 | Anti-Goat IgG (H+L) Cross-Adsorbed Secondary Antibody, Alexa Fluor™ 488 | Donkey | Invitrogen | A-11055 |
| 3 | Anti-Goat IgG (H+L) Cross-Adsorbed Secondary Antibody, Alexa Fluor™ 488 | Rabbit | Invitrogen | A-11078 |
| 4 | Anti-Human IgG-heavy and light chain, FITC conjugated | Goat | Bethyl | A80-119F |
| 5 | Human/Mouse/Rat/Hamster ACE-2 Antibody | Goat | R&D Systems | AF933 |
| 6 | Anti-Goat Alexa 594 | Rabbit | Immunotag | ITIF59418 |
| 7 | Anti-Goat IgG/HRP | Rabbit | Immunotag | ITSAH238 |
| 8 | MERS Coronavirus Spike Protein Polyclonal Antibody | Rabbit | Invitrogen | PA5-81786 |
